# Metabolic response after 68Ga-PSMA-PET/CT-directed IGRT/SBRT for oligometastases prostate cancer

**DOI:** 10.1007/s12094-022-03002-7

**Published:** 2022-11-11

**Authors:** Ahmed Gawish, Nurlan Abdullayev, Souhir El-Arayedh, Burkard Röllich, Hans-Joachim Ochel, Thomas B. Brunner

**Affiliations:** 1 Department of Radiation Oncology, University Hospital Magdeburg, Leipziger Str. 44, 39120 Magdeburg, Germany; 2 Department of Radiation Oncology, Medical University of Graz, 8036 Graz, Austria

**Keywords:** SBRT, Prostate cancer, Oligometastatic, PSMA-PET

## Abstract

**Background:**

We used 68Ga PSMA PET/CT in the current investigation to assess the metabolic response and local control of metastasis in patients with oligometastatic prostate cancer receiving SBRT.

**Materials and procedures:**

We performed a retrospective evaluation of the medical data of all patients with oligometastatic prostate cancer who underwent stereotactic body radiation therapy (SBRT) between 2017 and 2021. Our analysis only included medical records of patients who had SBRT for oligometastatic prostate cancer and had pre and post-SBRT 68Ga PSMA PET/CT images. Patient-related (age), disease-related (Gleason score, location of metastases), and treatment-related (factors and outcomes) data were collected from the medical files.

**Results:**

A total of 17 patients (28 lesions) with a median age of 69 years were included in the research. A median follow-up of 16.6 months was used (range 6–36 months). The median follow-up period for 68 Ga PSMA PET/CT was 8 months (the range was 5–24 months). The median pre-treatment PSA level was 1.7 ng/mL (range 0.39–18.3 ng/mL) compared to the post-treatment PSA nadir of 0.05 ng/mL (0.02–4.57). During the follow-up period, local control was 96%, and there was a link between PSMA avidity on PET. In the treated lesions, there were no recurrences. During follow-up, none of the patients experienced toxicities of grade 3 or above.

**Conclusions:**

SBRT is a highly successful and safe way of treating patients with oligometastatic prostate cancer. Additional research is needed to examine 68Ga PSMA PET/CT to assess further for demarcation and follow-up.

## Introduction

Treatment for patients with oligometastatic recurrence of hormone-sensitive prostate cancer (PCa) in the absence of local recurrence often consists of postponed androgen deprivation therapy (ADT) [1, 2]. Evidently, ADT has many adverse effects that can affect a person's quality of life. For example, it can cause hot flashes, fatigue, a decline in sexual functioning, problems with erections, loss of muscle and bone mass, and depression [3].

Stereotactic ablative radiotherapy (SABR) is a non-invasive treatment that provides good local control of localized tumor locations with minimal reported toxicity [4, 5]. Another benefit is that it just requires a short amount of time, as it is often given in 3–5 up to 12 high dose fractions [6].

Low-burden illness, along with advancements in imaging and radiation therapy (RT) delivery, has resulted in an increased utilization of ablative therapies for these patients. SABR-COMET [7], a randomized prospective phase II trial (21% prostate cancer), demonstrated a statistically significant survival advantage for stereotactic ablative radiation (median overall survival, 41 months versus 28 months in the SABR versus control group; HR 0.57), indicating that ablative approaches for oligometastatic disease should be further investigated.

Up to date, the outcome benefit of imaging and early diagnosis of metastatic diease is unknown. Novel positron emission tomography (PET) tracers enable the detection of oligometastases in patients with prostate cancer, both at the time of initial cancer diagnosis and during biochemical failure following therapy [8]. PSMA is a type II transmembrane protein that is generally overexpressed in prostate cancer tissue. PSMA PET/Computed Tomography (CT) with 68Gallium (68Ga) provides a good sensitivity for the diagnosis of low-burden metastatic illness [7].

Due to its non-invasive nature and short treatment duration, stereotactic body radiation (SBRT) is an attractive therapeutic option for metastasis-directed therapy. SBRT is used to give ablative radiation doses to limited size volumes with the goal of eradicating low-burden illness, improving local control, and, perhaps, survival or even cure [6]. However, there are few studies that have employed molecular imaging to measure the response after SBRT [9, 26]. We decided to retrospectively evaluate the use of 68Ga PSMA PET/CT to assess local control of bone and lymph node metastases in oligometastatic prostate cancer patients treated with SBRT in the current research.

### Material and methods

A retrospective evaluation of medical data of consecutive oligometastatic prostate cancer patients treated between 2017 and 2021 was done with consent from the institutional review board. Our study included only patients who had SBRT for oligometastatic lesions (defined as up to five metastases) and had pre- and post-SBRT 68Ga PSMA PET/CT images. Patient-related data (age), disease-related data (Gleason score, metastatic location), and treatment-related variables and outcomes were collected from medical files.

68-Ga-PSMA PET-CT scanning was performed utilizing a combined PET-CT procedure and a helical CT scanner. The PET images reconstructed for the fusion, the CT component involved the administration of oral and intravenous contrast media. Each patient was given ^68^Ga–PSMA by intravenous injection. About 60 min later, CT scans were collected from the vertex to the mid-thigh. A contrast-enhanced CT scan was performed 60 s after the injection of non-ionic contrast material (CM). Following that, an emission PET scan in 3D acquisition mode was performed for the same axial picture. The diagnostic CT images were utilized to fuse the PET data with the diagnostic CT images and to create an attenuation correction map. PET images were reconstructed using a line of response procedure with CT attenuation correction and shown on a computer workstation.

The activity of Ga 68–PSMA was evaluated by determining the maximal standardized uptake value (SUVmax). Positive results were evaluated for focally enhanced PSMA uptake that could not be explained by PSMA's normal biodistribution.

We analyzed and classified pre- and post-SBRT 68Ga PSMA PET/CT images to assess local control at the target volume [bone contained by the SBRT planning target volume (PTV) prescription dose to PTV (= in-field control)]. SBRT-induced local control/tumor response was categorized as follows: Complete response (CR) — post-treatment maximum standardized uptake volume (SUVmaxpost) ≤ the background SUV mean in normal bone (SUV-NB); partial response (PR) — SUVmaxpost was smaller than the prior maximum SUV (SUVmaxpre), but more than the SUV-NB. (c) No Response (NR) – SUVmaxpost was larger than or equal to SUVmaxpre, according to PERCIST criteria response [10].

Our departmental SBRT OMD metastases protocol entailed the following: in a vacuum bag, an IV contrast-enhanced planning CT was conducted with a slice thickness of 1.0– 2 mm, followed by a 4D-CT for respiratory motion management.

To delineate the target, 68 Ga PSMA PET/CT was fused to the planning CT. The gross tumor volume (GTV) was manually segmented using attenuation corrected PSMA images overlaid on full dose CT, which provides anatomical boundaries for PET-positive areas [11]. The clinical target volume (CTV) for vertebral lesions was segmented according to ISRC criteria [12]. For non-spinal bony or lymph node lesions, the PTV was defined as a 5 mm extension of the GTV surrounding the lesion. Five to twelve fractions were prescribed for 17 patients with 28 lesions (Table 1) The RT was delivered, guided by Megavolt CT (MVCT) and orthogonal pictures prior to each fraction.

**Table 1:** Characters of the patients

	Nr	Percent
Patient	17/17	100%
Lesions	28/28	100%
Lymph node	21/28	75%
Presacral	3/28	10.7%
Perirectal	6/28	21.5%
Iliacal	9/28	32.1%
Para-aortal	3/28	10.7%
Bone	7/28	25%
Vertebra	3/28	10.7%
Rib	3/28	10.7%
Os ilium	1/28	3.6%
pT3	14/17	50%
pN1	3/17	10.7%
R1	4/17	14.2%
Prior Radiation Prostatic fossa	8/17	28.5%
PSA Nadir post SBRT	0–1.27	n.a
Mean	0.262	n.a
Median	0.1275	n.a
PSA before RT	0.39–18.3	n.a
Mean	1.07	n.a
Median	3.956	n.a
SUVmax before SBRT	2.6–58	n.a
Mean	15.65	n.a
Median	8.3	n.a
SBRT courses	17	

Different SBRT regimens were utilized depending on the target dimension or metastasis’s location. When dose restrictions for organs at risk were not satisfactory, either replanning or a hypofractionation fractionation schedule was considered. SBRT, defined as by Guckenberger et al. [13], in 1–12 fractions was employed in 67% of the patients and hypofractionation in 33%.

Our follow-up plan involved a clinical examination (history and physical exam) every six months, as well as serum PSA testing. Repeat 68Ga PSMA PET/CT scans were done on patients with increasing PSA following SBRT to re-stage the patients prior to treatment selection, and in certain cases to determine response to therapy in the absence of evidence of biochemical failure.

**Fig. 1:**
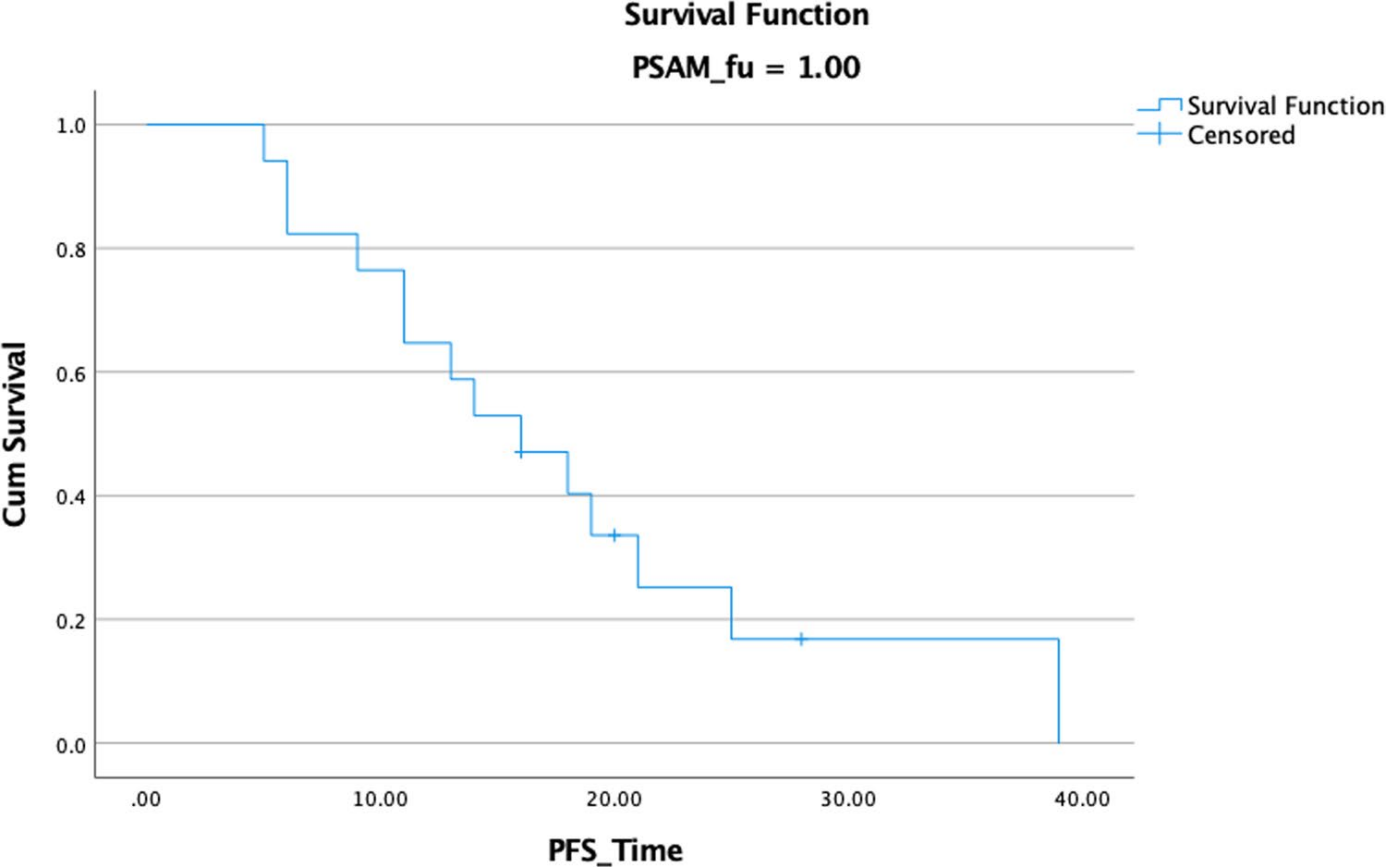
bFS in 17 patients with 28 lesions treated with SBRT for oligorecurrent prostate cancer

The median and range were used to convey quantitative aspects, whereas frequency and percentage were used to describe qualitative characteristics. The statistical analysis was performed using SPSS 20.0 program (SPSS for Window, IBM Corp., Armonk, NY, USA). Actuarial rates of LC, Biochemical-free survival (bFS) (Fig. 1), and overall survival were calculated using Kaplan–Meier analysis (OS). We collected the SUV max from our PACS system (INFINIT). Log-rank testing was used to ascertain the relationship between patient-related variables and treatment outcomes. The parameters' Pearson correlations were found. A p-value of less than 0.05 was used to determine statistical significance.

## Results

**Fig. 2:**
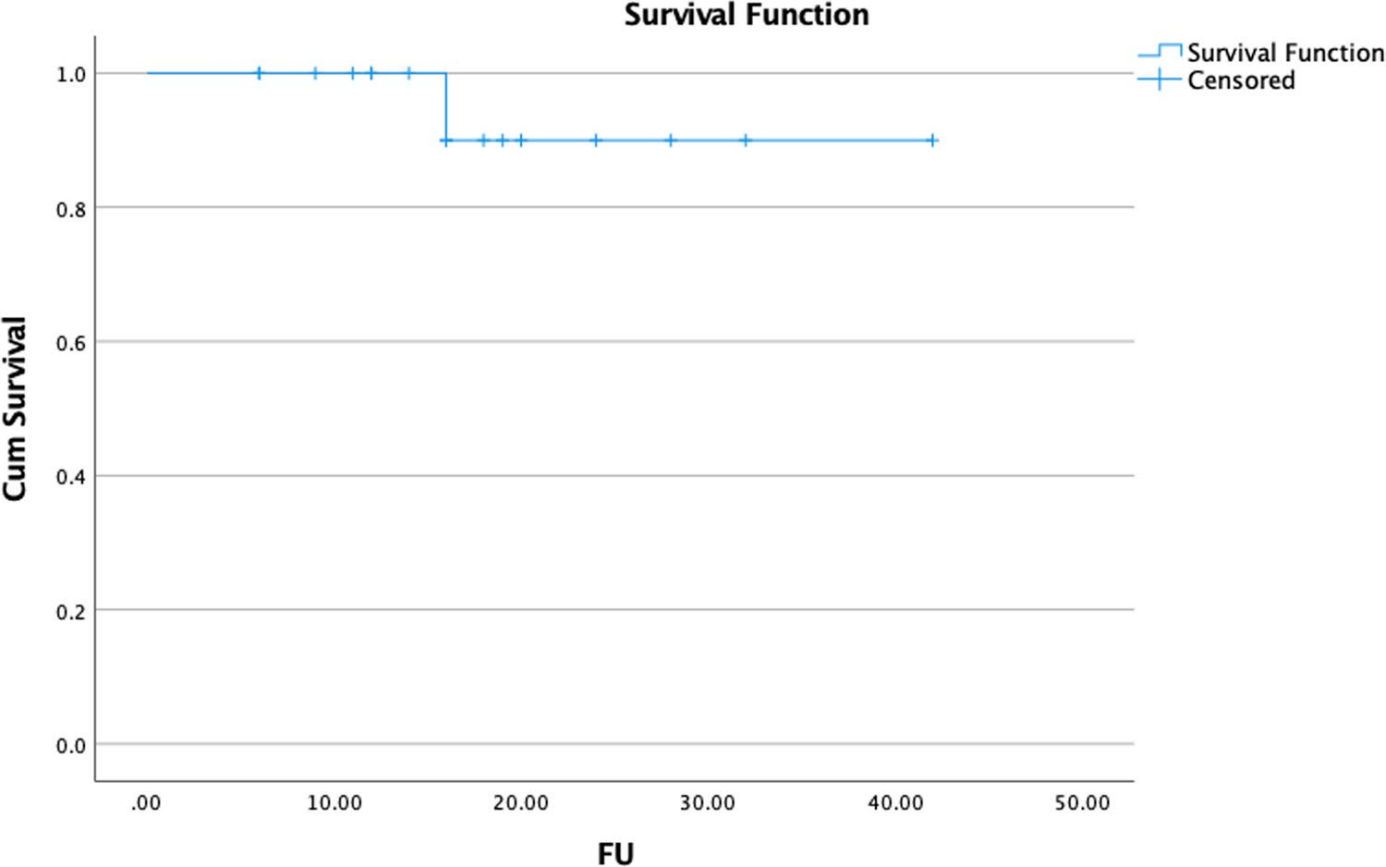
Metabolic local control in 17 patients with 28 lesions treated by SBRT for oligometastatic prostate cancer

Throughout the research period, a total of 17 patients with 28 lesions (21 lymph nodes and 7 bone) with a median age of 69 years were enrolled. All patients had radical prostatovesiculectomy prior to SBRT (Fig. 2). The median interval between follow-up visits was 16.6 months (range 6–36 months). The median interval between SBRT and re-staging by 68 Ga PSMA PET/CT was 8 months (range 5–24 months). Table 1 summarizes the characteristics of patients and treatments.

**Fig. 3:**
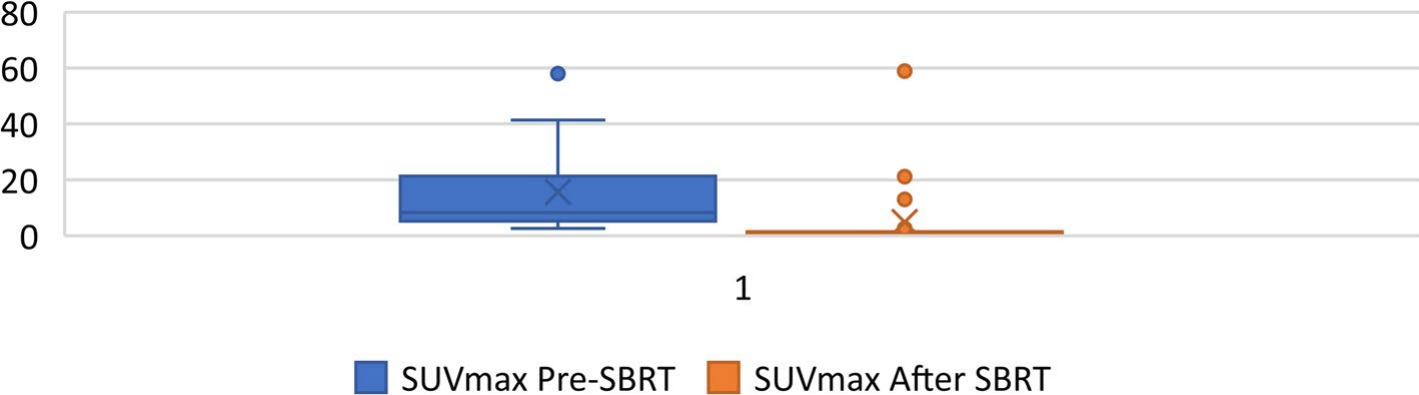
SUVmax Pre- and Post-SBRT

During the follow-up, none of the patients died. The median pre-treatment PSA level was 1.7 ng/mL (range 0.39–18.3 ng/mL), whereas the median post-treatment PSA level was 0.05 ng/mL (0.02–4.57). SUVmax pre-treatment median value was 8.3 (range 2.6–58). On repeat PSMA PT/CT, complete response was found in 27 of 28 (96.4 percent) lesions, with a median SUVmax-pre-RT of 8.3 (2.6–58) decreasing to 1.1 SUVmax-post RT (range 0–58) (p 0,001). (Fig. 3). Of the 28 lesions, 27 lesions had a metabolic response on PSMA PET with a drop in SUVmax of at least 50% and a substantial reduction in lesion size; in eight of these lesions no uptake of 68Ga-PSMA was detected with SUV values below the threshold on follow-up PET/CT. One lesion (Lymph node) exhibited persistent (SD) PSMA avidity 5 months after SBRT but without correlation in CT, maximum diameter decreased from 23 to 12 mm, while SUVmax increased from 37.5 to 58.

**Fig. 4:**
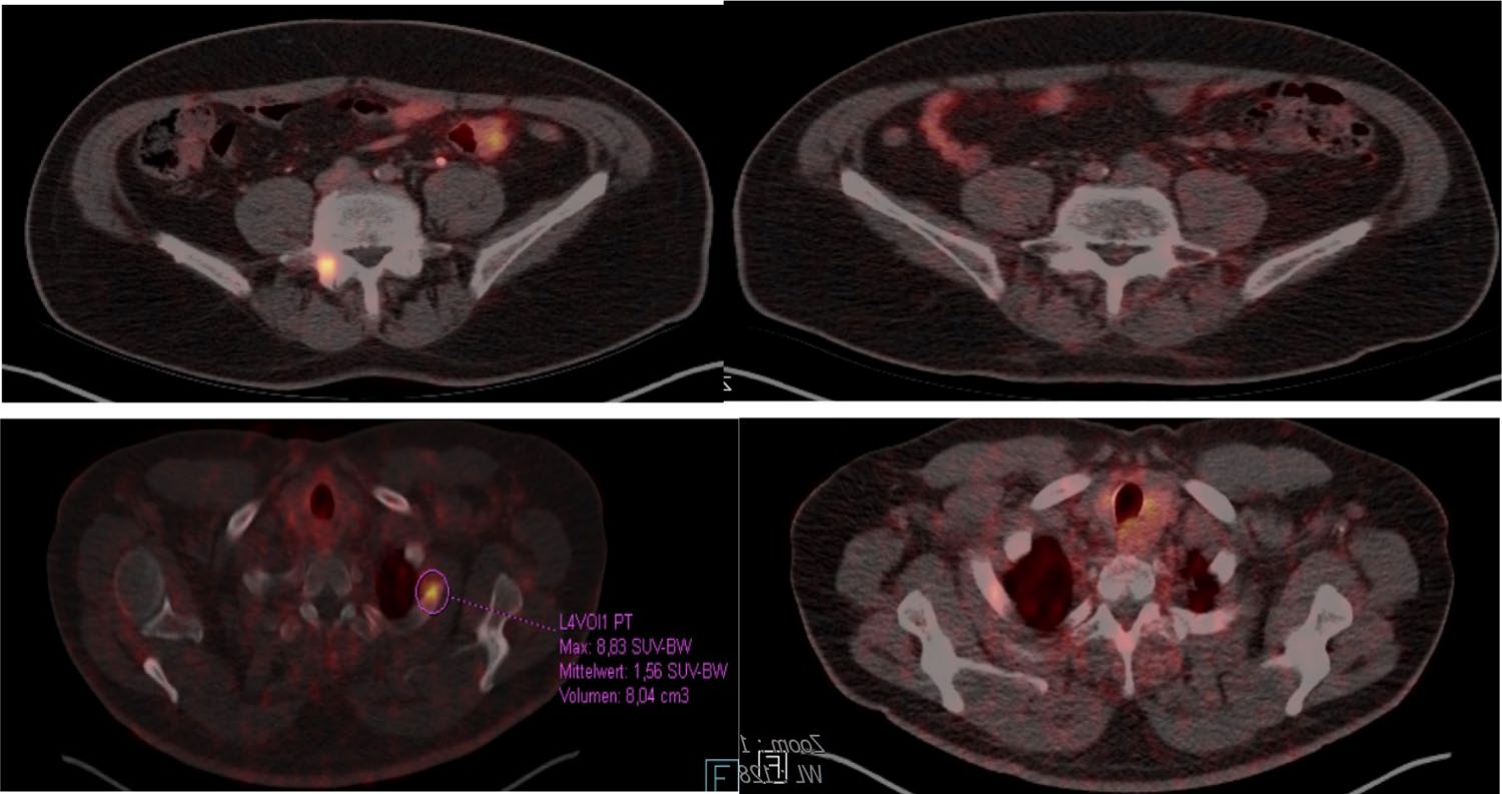
Example PSMA PET before and after SBRT

The mean delay between the termination of radiotherapy and the follow-up PET/CT was 3 months (range 1.9–15 months). Repeated 68 Ga PSMA PET/CT scans pre- and after SBRT are shown in Fig. 4, exhibiting diminishing avidity with time. On follow-up PET/CT, one of the treated lesions progressed with an increase in SUVmax > 20%. (One lymph node metastasis).

Mean size of the PSMA-GTV was 2.69 mL (median 1.26; range 0.22–12.3) and mean PTV was 14.9 mL (median: 10.32; range 2.54–41). A total of 10 treatments were with SIB-concept adding an elective treatment of the nodal region to a median EQD2_2_ of 50 Gy. Mean dose was 46.8 Gy (median 48; range 30–60) in a mean of 12 fractions (median, 12 range 5–20), with a mean daily dose of 4.6 Gy (median 4; range 3–8 Gy). Mean BED_2_ was 140.5 Gy (range 105–153), mean EQD2_3_ was 70 Gy and mean EQD2_10_ was 56.3 Gy.

### Recurrences

On all consecutive PSMA PET/CT scans, new metastatic lesions were found. All lesions occurred outside of the locations that had been irradiated. Previously, no escalation of the irradiation volume following a metabolic reaction has been reported. 6 Patients received second session on SBRT, 8 Patients received ADT and two received radiotherapy to prostatic fossa and only one patient started with docetaxel (Table 2).

**Table 2:** Outcome and further course post SBRT

Clinical outcome		
Local recurrence in SBRT volume	0/17	
Out of field		
BCF without + ve lesion ()		
Biochemical control	1	
Second SBRT	5/17	4 LN and 1 Bone
RT prostatic fossa	2/17	
ADT	8/17	
Chemotherapy	1/17	
No therapy	1/17	
Metabolic outcome		
CMR	27	
PMR	0	
SMD	1/28	

After one-year, local metabolic control (using metabolic response as a surrogate for local control) was 96% percent at irradiated sites; after one-year, local control was 100 percent for lesions treated with SBRT to a median dosage of BED 10 > 67 Gy.

### Toxcity

All patients underwent the complete course of therapy, which was generally well tolerated and associated with a low incidence of adverse effects. In an acute context, 100% of patients suffered side effects of grades 1–2. Most often mentioned side symptoms were nausea and exhaustion. No patient experienced grade 3 or 4 side effects.

## Discussion

Our study demonstrates that SBRT is an effective treatment option for oligometastases prostate cancer. The difference in SUVmax between post- pre-PSMA PET was used to recognize the dramatic local response and metabolic regression of bone and lymph node metastases and was associated with clinical outcome (patient's clinical evaluation).

Despite the limited size of our cohort, this study highlights many clinically significant issues. SBRT targeting the OMD resulted in metabolic complete remission in 96% of irradiated sites, similar rates were reported by Ost [4]. None of the patients developed a grade 4 toxicity or suffered from treatment-related problems in the treated lesions, no patient needed re-treatment in the SBRT-field. All patients were still alive after a median follow-up of 16 months, indicating that this patient population should be administered treatment that offers good local control.

The lesions discovered and treated in this work were rather small, with median and mean volumes of 1.26 and 2.63 cm^3^, respectively. According to data from prior studies, PSMA PET has a significantly greater detection rate than all other currently available imaging modalities [14, 15]. It is proven that a significant proportion of these lesions would have gone undetected during standard staging procedures. Thus, PSMA PET appears to be an effective tool for treatment planning in patients with oligometastatic prostate cancer planned for local ablative therapy. A significant difficulty for patients in whom PSMA PET is utilized to plan radiation is defining the treatment target volume. This is critical, even more so for patients planned to get high-precision radiation. In the case of primary prostate cancer, PSMA PET appears to overestimate tumor volume when compared to magnetic resonance imaging (MRI) [16]. The PET-positive volume was utilized to define the target volume qualitatively at window settings used for diagnostic imaging, and no complementary imaging techniques were regularly employed to further define the size of treated lesions. Although overestimating the target volume cannot be ruled out, it is unlikely to occur in a single, well-defined lymph node. However, defining the target may be more challenging in bony structures. PSMA PET may underestimate, hypothetically, microscopic tumor expansion in the bone marrow, for example, in the iliac bone or vertebra. As a result, a small safety margin was established to account for such microscopic expansion. The first findings of this inquiry indicate that this strategy may be appropriate for routine clinical practice.

Furthermore, we found a significant metabolic response rate for PSMA PET-detected lesions following hypo-fractionated radiation. After SBRT, 60% of all lesions were no longer identifiable according to PERCIST criteria [17]. It is difficult to compare CR results to earlier studies with traditional fractionation in terms of response evaluation due to the use of various criteria and imaging (if any) (e.g., re-treatment, pain relief). Additionally, morphological characterization of bone and lymph node metastases using other imaging techniques might be significantly less sensitive. PSA levels and routine CT imaging are used to assess response in prostate cancer patients using the response evaluation criteria in solid tumors (RECIST) criteria [14].

Prostate cancer bone metastases typically result in an irreversible osteoblastic response that may or may not include live cancer cells, making them challenging to diagnose using RECIST [10]. As demonstrated in our investigation, the use of molecular imaging with 68Ga PSMA PET/CT should be further studied as a novel tool for planning the target volume and evaluating response to SBRT [16].

SBRT may offer a novel therapeutic option for men with oligorecurrent prostate cancer, slowing disease development and avoiding the side effects associated with systemic therapy. While numerous research reports on the clinical result of local irradiation using "traditional" imaging techniques such as MRI or choline PET [18–20], just a few reports on PSMA-guided metastasis guided radiotherapy. According to research performed by a group from Heidelberg [21], 83.1 percent of patients with oligorecurrent prostate cancer who underwent PSMA PET/CT had a PSA response following local irradiation. This is consistent with a study of 83 patients with biochemical recurrence following surgery, which found that PET guided, fractionated radiation for nodal relapses lowered PSA in 82.9 percent of patients [22]. Interestingly, the superior local control rates following irradiation were highly correlated with a reduction in SUVmax in those patients having follow-up PSMA imaging.

The STOMP study [4], asymptomatic PCa patients who presented with up to three extracranial lesions on choline PET-CT and serum testosterone levels greater than 50 ng/ml were eligible for this trial in the event of a biochemical recurrence after primary PCa therapy with curative intent. All identified lesions were randomly assigned (1:1) to either surveillance or MDT for patients. Two factors, PSA doubling time (3 vs. > 3 months) and nodal vs. non-nodal metastases, were dynamically adjusted during randomization. Local treatment or surveillance was assigned to patients, and the main objective was androgen deprivation therapy-free survival. Local treatment greatly delayed the need for androgen deprivation therapy, which was necessary in the event of symptomatic progression, the incidence of more than three metastases, or the local advancement of a known metastasis. The ORIOLE trial [22] allocated males with up to three metastases diagnosed with conventional imaging to observation or SBRT for all detected metastases following additional assessment with PSMA PET imaging. At six months, 60% of men who were observed had progressed, compared to just 19% of men who had SBRT. Mazolla et al. [23] conducted a retrospective examination of PSMA PET guided vs choline PET-guided SBRT, indicating that PSMA control was greater with choline PET guided SBRT. These findings imply that PET PSMA-guided metastasis-directed treatment may be beneficial in individuals with oligometastatic prostate cancer. This method may help delay the need for androgen deprivation, which may result in enhanced quality of life and a delay in the development of androgen independent illness. The Oriole trial biomarkers indicate that SBRT triggered a systemic immune response, necessitating additional investigation of this technique.

Numerous limitations apply to the current investigation. This is a retrospective study with a short follow-up period, In addition, the it comprised a very small number of patients, which diminishes the validity of the analysis. It varies from the STOMP and ORIOLE studies insofar that eight of twelve patients underwent 3 months of short-term androgen deprivation concomitant with SBRT that was terminated upon radiation completion.

Finally, hypo-fractionated IGRT with BED10 > 67 is a treatment regimen that has been shown to be effective in the treatment of low-risk primary prostate cancer [24]. Other Institutes have also utilized this regimen to treat metastases [25]. The data presented here demonstrate that SBRT is promising, even for metastatic locations, and is associated with a high rate of success and tolerability (Table 3).

**Table 3:** Radiotherapy dose regimes

Dose regime		BED_2_	BED_3_	BED_10_	EQD2_2_	EQD2_1,5_	EQD2_3_	EQD2_10_
48/4 Gy	20/52 (38%)	144	112	67.2	72	75	67.2	56
60/3 Gy	16/52 (31%)	150	120	78	75	77.14	72	65
35/7 Gy	8/52 (15%)	157.5	116.67	59.5	78.75	85	70	49.58
30/6 Gy	2/52 (4%)	105	80	45	52	64.3	48	37.5
40/5 Gy	2/52 (4%)	140	106.6	60	70	74.3	64	50
40/4 Gy	4/52 (8%)	144	112	67.2	60	62.86	56	46.7

In summary, the early findings indicate that PSMA PET-detected metastatic lesions can be efficiently treated with high-precision radiation directed at the PSMA PET-positive tumor volume. Further research of the metabolic response in follow-up PSMA PET/CT is required. If PSMA PET is utilized to assess response, a period of many months following radiation may be necessary to accurately predict treatment efficacy.

## Conclusions

SBRT is a highly successful and safe way of treating patients with oligometastatic prostate cancer. Additional research is needed to examine 68Ga PSMA PET/CT assess further for demarcation and follow-up. SBRT alone or in conjunction with short-term ADT should be explored further to determine the appropriate treatment to oligometastatic recurrence.

## Data Availability

The datasets used and analyzed during the current study are available from the corresponding author on reasonable request.

## Abbreviations

RP: Radical prostatectomy
RT: Radiotherapy
SBRT: Stereotactic body radiation therapy
OMD: Oligometastatic disease
ADT: Androgen deprivation therapy
ECOG: Eastern Cooperative Oncology Group
OS: Overall Survival
PFS: Progression free survival
BCF: Biochemical Failure
IGRT: Image guided radiation therapy
PERCIST: Positron Emission Tomography Response Criteria in Solid Tumors
PCa: Prostate cancer
