# Correction to: Metabolic response after 68 Ga‑PSMA‑PET/CT‑directed IGRT/SBRT for oligometastases prostate cancer

**DOI:** 10.1007/s12094-023-03104-w

**Published:** 2023-02-18

**Authors:** Ahmed Gawish, Nurlan Abdullayev, Souhir El-Arayedh, Burkard Röllich, Hans-Joachim Ochel, Thomas B. Brunner

**Affiliations:** 1 Department of Radiation Oncology, University Hospital Magdeburg, Leipziger Str. 44, 39120 Magdeburg, Germany; 2 Department of Radiation Oncology, Medical University of Graz, 8036 Graz, Austria

**Correction to: Clinical and Translational Oncology** 10.1007/s12094-022-03002-7

In this article the author names Nurlan Abdullayev and Souhir El-Arayedh were incorrectly written as Nurlan Abdulayev and Souhir El-Arayed.

The original article has been corrected.

